# MEDUSA: A cloud-based tool for the analysis of X-ray diffuse scattering to obtain the bending modulus from oriented membrane stacks

**DOI:** 10.1371/journal.pcbi.1011749

**Published:** 2024-01-08

**Authors:** Sebastian Himbert, Dorian Gaboo, Emre Brookes, John F. Nagle, Maikel C. Rheinstädter

**Affiliations:** 1 Department of Physics and Astronomy, McMaster University, Hamilton, Ontario, Canada; 2 Origins Institute, McMaster University, Hamilton, Ontario, Canada; 3 Department of Chemistry and Biochemistry, University of Montana, Missoula, Montana, United States of America; 4 Department of Physics, Carnegie Mellon University, Pittsburgh, Pennsylvania, United States of America; University of Virginia, UNITED STATES

## Abstract

An important mechanical property of cells is their membrane bending modulus, *κ*. Here, we introduce MEDUSA (**ME**mbrane **D**iff**U**se **S**cattering **A**nalysis), a cloud-based analysis tool to determine the bending modulus, *κ*, from the analysis of X-ray diffuse scattering. MEDUSA uses GPU (graphics processing unit) accelerated hardware and a parallelized algorithm to run the calculations efficiently in a few seconds. MEDUSA’s graphical user interface allows the user to upload 2-dimensional data collected from different sources, perform background subtraction and distortion corrections, select regions of interest, run the fitting procedure and output the fitted parameters, the membranes’ bending modulus *κ*, and compressional modulus *B*.

## 1 Introduction

Cellular functions, such as mobility, division and vesicle trafficking, are intrinsically related to a cell’s ability to comply to deformation [1–3]. Especially the cell membrane’s endurance against bending forces is critical for a cell’s survival. The Helfrich Hamiltonian [4] for the energy of a bent symmetric membrane is:

$$\begin{array}{c} E=\frac{\kappa }{2}\int_{A}da{\left(\nabla^{2}u\left(\vec{r}\right)\right)}^{2}, \end{array}$$ (1)

where $u(\vec{r})$ describes local spatial deviation of the bilayer center in the out-of-plane direction (with respect to the membrane) and the integral is over the area covered by the membrane. The bending modulus *κ* is a material property. As such, it varies with temperature and the molecular composition of the membrane and is a particularly appropriate measure of overall membrane elasticity.

Various techniques have been developed to study cell membrane elasticity. Mechanical properties on cellular length scales were measured by micropipette aspiration [5], while atomic force microscopy [6] probes elastic behavior on the nanoscale. Cell stiffness is also studied indirectly by spectral analysis of flickering of cell membranes under a microscope [7–9], as well as optical interferometric techniques [10, 11]. Methods that probe the bending modulus on smaller length-scales (<80 nm) include X-ray diffuse scattering (XDS) [12–16], neutron spin echo (NSE) spectrometry [15, 17], NMR [18] and MD simulations [15, 19]. This paper focuses on the XDS method.

In XDS experiments, the sample consists of stacks of solid supported multilamellar membranes and the X-ray scattering is measured. Such samples are widely used to probe the structure of synthetic lipid bilayers [20–24] as well as more complex biological membranes [25, 26]. Importantly, such samples are ideal to analyze the mechanical properties of these molecular structures as it was first introduced by Lyatskaya et.al [12] and has been subsequently applied to single [13, 14, 27] and multi-component [28–30] lipid bilayers culminating in studies on native red blood cell membranes [15, 16, 31]. Advancements in comprehending the molecular-level mechanics within these membranes have given rise to a variety of sophisticated techniques, facilitating the utilization of endogenous membranes for various biotechnological applications [32, 33].

**Fig 1:**
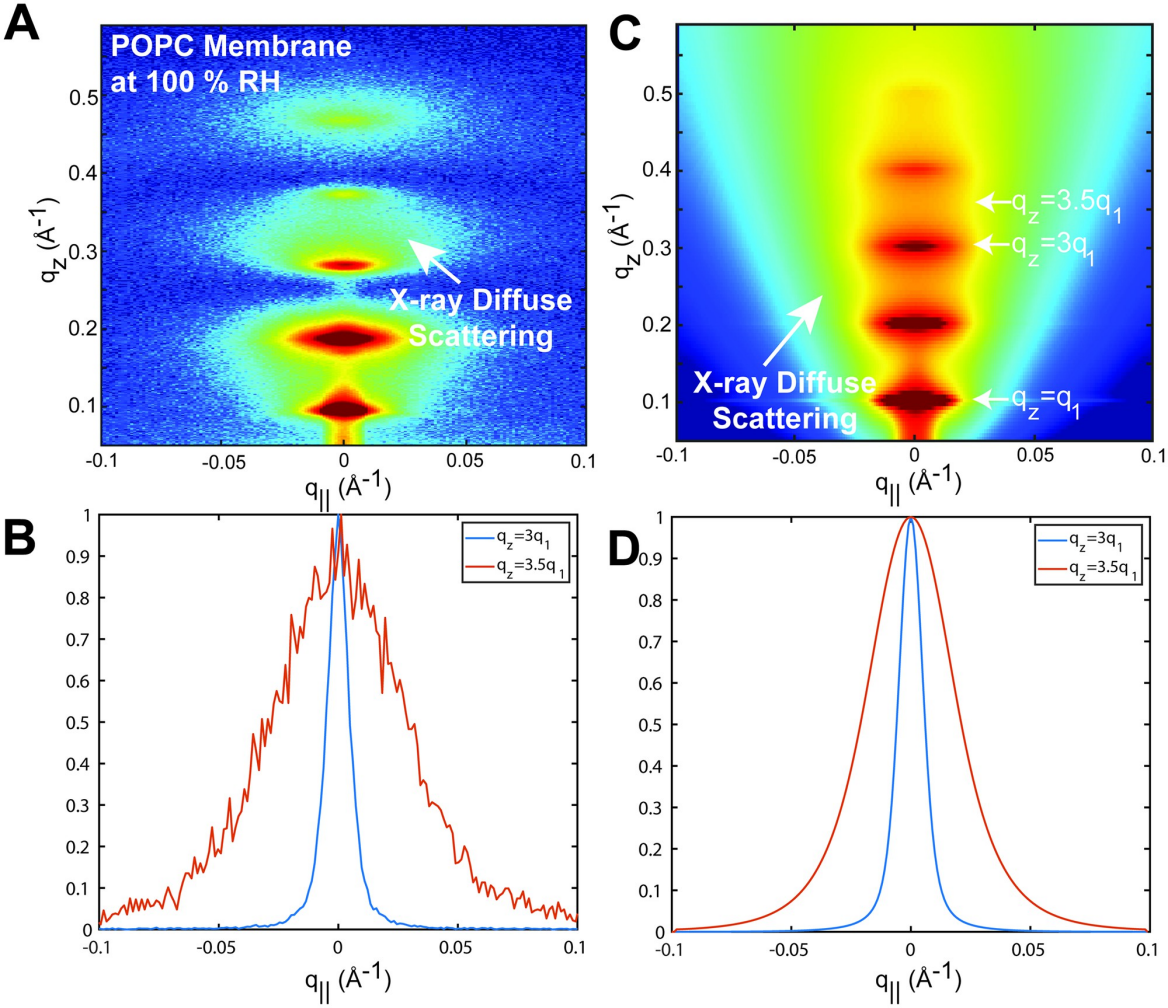
**A** 2-dimensional X-ray intensity map recorded on a stack of POPC bilayers. Measurements were performed 100% RH. The most intense scattering is specular (*q*_‖_ = 0) with additional diffuse off-specular scattering that originates from out-of-plane membrane fluctuations. **B** Intensity profile of A along the out-of-plane scattering vector *q*_‖_ at *q*_*z*_=3*q*_1_ and 3.5*q*_1_, where *q*_1_ refers to the 1^*st*^ order lamellar peak. **C** 2-dimensional map of Eq 4. **D** Simulated line-cuts of C at *q*_*z*_=3*q*_1_ and 3.5*q*_1_.

An exemplary result is shown in Fig 1A. The most intense scattering is specular (*q*_‖_=0 Å^−1^) with peaks that originate from the average lamellar repeat distance in the stack of membranes, and it includes the sharp line of reflectivity from the solid support. The space between neighboring membranes is occupied by water and the thickness *d*_*w*_ of this water layer is controlled by the environments relative humidity (RH). In experiments, *d*_*w*_ is commonly calculated as *d*_*w*_ = *d* − *d*_*m*_, where *d* represents the lamellar repeat spacing and *d*_*m*_ is the membrane thickness (also known as Luzzati thickness) as defined in [34]. *d*_*w*_ is small (*d*_*w*_ ≈ 13 Å for RBC membranes [25]) below 98% RH and out-of-plane membrane fluctuations are suppressed. Well hydrated samples (>99.9% RH), on the other hand, have a significantly larger (*d*_*w*_ ≈ 28 Å) water layer between the membranes [15], enabling out-of-plane fluctuations of the individual membranes. This results in an additional diffuse off-specular scattering (see Fig 1B). Membrane fluctuations are thermally driven and their amplitude and spectrum vary with the bending rigidity *κ*. The intensity in the off-specular regime is closely related to the membrane structure factor as will be discussed in depth below, so one can experimentally determine *κ* by fitting the model structure factor as a function of *κ* to the experimental out-of-plane intensity profile.

Calculating the structure factor is computationally challenging. Here, we introduce MEDUSA (**ME**mbrane **D**iff**U**se **S**cattering **A**nalysis), a cloud-based analysis tool to determine the bending modulus, *κ*, from XDS experiments. MEDUSA uses GPU accelerated hardware and a parallelized algorithm to run the calculations efficiently. MEDUSA’s graphical user interface allows the user to upload 2-dimensional data collected from different sources, perform background subtraction and distortion corrections, select regions of interest, run the fitting procedure and output the fitted parameters, the membranes’ bending modulus *κ* and interaction modulus *B*. The tool is available at https://medusa.genapp.rocks.

As diffuse scattering is typically orders of magnitude weaker than specular scattering, these measurements require X-ray sources with sufficient intensities. Both synchrotron sources and optimized rotating anode in-house machines have been used. A critical element of the experimental design is the humidity controlled sample environment as measurements need to be performed close to 100% RH. Various designs have been developed in the past. We will describe two successful chamber designs and provide insight into the challenges and caveats.

## 2 Design and implementation

### 2.1 Membrane scattering theory

The intensity measured in an X-ray diffraction experiment can be written as

$$\begin{array}{ccc} \left\langle I\right\rangle & = & |F\left(q_{z}\right){|}^{2}\left\langle S\left(\vec{q}\right)\right\rangle , \end{array}$$ (2)

where *F*(*q*_*z*_) is the form factor and $S(\vec{q})$ is the structure factor. In the case of solid supported lipid bilayer, this structure factor has the form [12]:

$$\begin{array}{ccc} S(q_{z},q_{\|}) & = & \sum_{n=-\infty }^{n=\infty }H_{z}\left(nd\right)\;\text{cos}\left(q_{z}nd\right)\\ & \times & \int_{0}^{\infty }rdrH_{r}\left(r\right)J_{0}\left(q_{\|}r\right)e^{iq_{z}\delta u_{n}\left(r\right)}\text{,} \end{array}$$ (3)

where *J*_0_ is the zero order Bessel function and *δu*_*n*_(*r*) is the height-height pair correlation function. The functions *H*_*z*_(*z*) and *H*_*r*_(*r*) account for experimental limitations that reduces the effective size of coherently scattering membrane patches. A limiting factor in the out-of-plane direction is the number of membranes that can be stacked with a sufficient degree of order. The in-plane direction is limited by the sample’s dimensions, the footprint of the beam (typically in the order of <200 *μ*m) on the sample, but most importantly, by the diameter of coherently scattering membrane patches. These patches are different from the lipid domains that occur in mixed lipid bilayers and need to be understood as regions that scatter coherently and are finite due both to finite beam coherence and to sample mosaicity. The membrane stack then consists of many of these regions. The structure factor for such a patchy sample has been described in detail in [12] and [35]. Briefly, they are assumed to be cylindrical patches with a Gaussian distributed diameter *L*_*r*_ (average patch size *L*_*r*_ and variance *σ*_*r*_) and a Gaussian distributed height *L*_*z*_ (average patch size *L*_*z*_ and variance *σ*_*z*_) and define size effect functions [12]:

$$\begin{array}{ccc} H_{z}\left(z\right) & = & \int_{z}^{\infty }dL_{z}\frac{1}{\sigma_{z}}\;\text{exp}\;(-\frac{{\left(L_{z}-\bar{L_{z}}\right)}^{2}}{2\sigma_{z}^{2}})\frac{\left(L_{z}-z\right)}{d}, \end{array}$$ (4)

$$\begin{array}{cc} H_{r}\left(r\right) & =\int_{r}^{\infty }dL_{r}\frac{1}{\sigma_{r}}\;\text{exp}\;(-\frac{{\left(L_{r}-\bar{L_{r}}\right)}^{2}}{2\sigma_{z}^{2}})L_{r}^{2}\\ & \times \left\{ \begin{array}{cc} 0 & \frac{r}{L_{r}}>1\\ {\text{cos}}^{-1}(\frac{r}{L_{r}})-\frac{r}{L_{r}\sqrt{1-{\left(r/L_{r}\right)}^{2}}} & \frac{r}{L_{r}}\leq 1. \end{array}\right. \end{array}$$ (5)

XDS is the off-specular scattering observed in the diffraction experiment. Since *F*(*q*_*z*_) only depends on *q*_*z*_, by Eq 2, diffuse scattering is solely governed by the structure factor *S*(*q*_*z*_, *q*_*r*_) which depends on the inter- and intra-lamellar height-height pair correlation function *δu*_*n*_(*r*) of the membrane stack. This is shown in Fig 1C which depicts a 2-dimensional map of Eq 4 and illustrates the out-of plane contribution (Fig 1D). A non-vanishing *δu*_*n*_(*r*) arises from thermally excited out-of-plane fluctuations; an analytical expression has been derived from the stack’s free energy [12].

### 2.2 Stack model

Eq 1 describes the energy of a single lipid bilayer. For a sample consisting of a stack of bilayers, an interaction between bilayers must be added [12, 36]:

$$\begin{array}{c} \begin{array}{c} F=\int_{A}d^{2}r\sum_{n=0}^{N-1}(\frac{1}{2}\kappa {\left(\nabla_{r}^{2}u_{n}\left(\vec{r}\right)\right)}^{2}\\ +\frac{1}{2}\frac{B}{d}{\left(u_{n+1}\left(\vec{r}\right)-u_{n}\left(\vec{r}\right)\right)}^{2})\text{,} \end{array} \end{array}$$ (6)

where *B* is a modulus that accounts for the interaction between neighboring membranes in the harmonic approximations. [37].

Calculating the height-height pair correlation function *δu*_*n*_(*r*) from Eq (6) has been described in detail in [37]. Briefly, membrane fluctuations are governed by thermal energy and can be separated into normal modes by transforming the out-of-plane displacement $u_{n}\left(\vec{r}\right)$ into Fourier space ($u_{n}\left(\vec{r}\right)\to U_{n}\left(\vec{Q}\right)$). $\vec{Q}$ spans the Fourier space of the membrane fluctuations and differs form the scattering vector $\vec{q}$. The free energy functional in Eq (6) decouples in Fourier space. The equipartition theorem then assigns $\frac{1}{2}\;k_{B}T$ of energy to each normal mode. This allows calculating the power spectrum of the membrane fluctuations.

In Fourier space, the height-height pair correlation function is proportional to this power spectrum and an analytical expression of $\delta u_{n}\left(\vec{r}\right)$

$$\begin{array}{c} \delta u_{n}\left(\vec{r}\right)=\frac{2\eta_{c}}{q_{1}^{2}}\int_{0}^{\infty }dx\frac{1-J_{0}\left(r/\xi \sqrt{2x}\right){\left(\sqrt{1+x^{2}}-x\right)}^{2n}}{x\sqrt{1+x^{2}}} \end{array}$$ (7)

was derived [37], where *J*_0_ is the zero order bessel function, *q*_1_ = 2*π*/*d*, and *ξ* and *η* are known as Caillé parameters which relate to the bending modulus *κ* and the membrane interaction modulus B through [12]

$$\begin{array}{c} \eta_{c}=\frac{k_{B}Tq_{1}^{2}}{8\pi \sqrt{B\kappa }}\;\text{and}\;\xi^{4}=\frac{\kappa }{B}. \end{array}$$ (8)

Here, *k*_*B*_ is the Boltzmann constant and *T* is the temperature. One can determine both, the bending modulus *κ* and the interaction modulus *B*, independently by fitting the structure factor *S*(*q*_*z*_, *q*_‖_) to experimental data.

### 2.3 Data processing

MEDUSA is built using the GenApp framework [38]. GenApp enables a convenient web deployment of code in any programming language. Details on GenApp can be found elsewhere [38] and the following paragraphs solely focus on the two back-end programs.

### 2.4 Reduction

**Fig 2:**
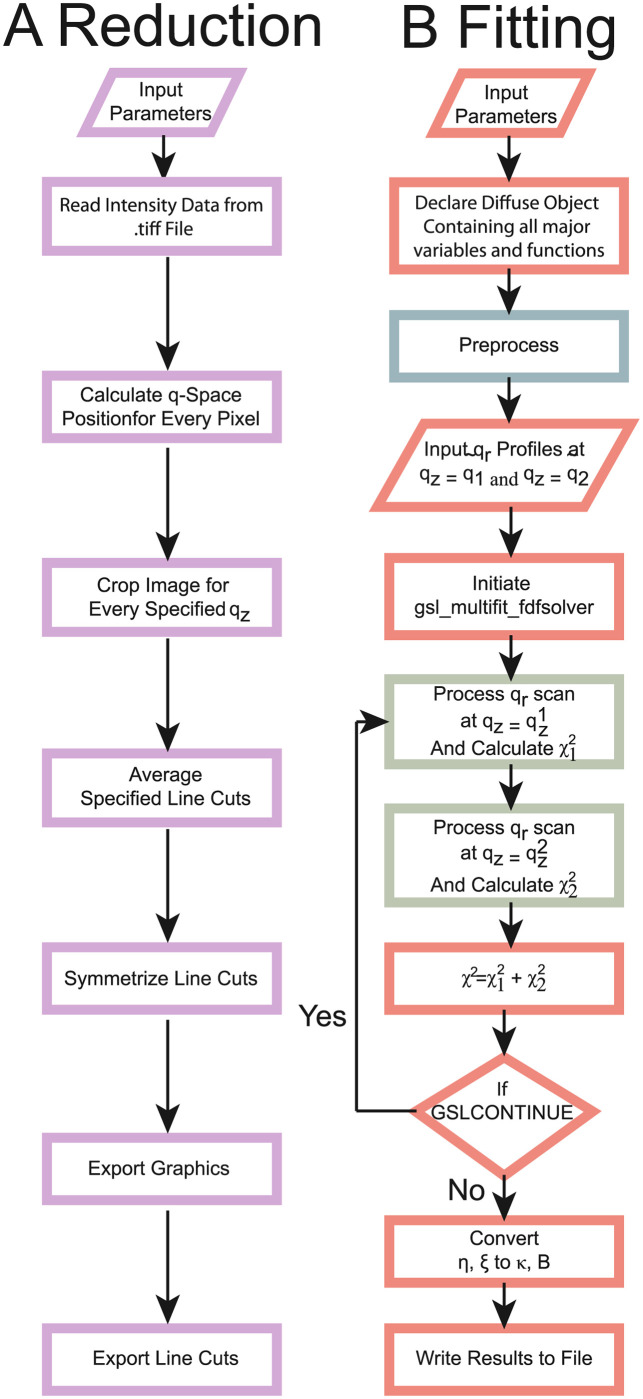
**A**) Flow diagram of the reduction routine (written in Python). **B** Flow diagram of the fitting program (written in C++ and CUDA). Critical subroutines are highlighted in light blue and light green, and are visualized in greater detail in Fig 4.

MEDUSA supports 2-dimensional intensity maps in the *Tag Image File Format* (.*tiff*) file-format. In a first step, data are loaded and reduced by a reduction library developed in Python [39]. The program flow of the data reduction is visualized in Fig 2A. Intensities can be stored on a linear or logarithmic scale. A rudimentary background-subtraction routine is implemented as discussed below. However, it is recommended to subtract any instrumental background for optimal results.

**Fig 3:**
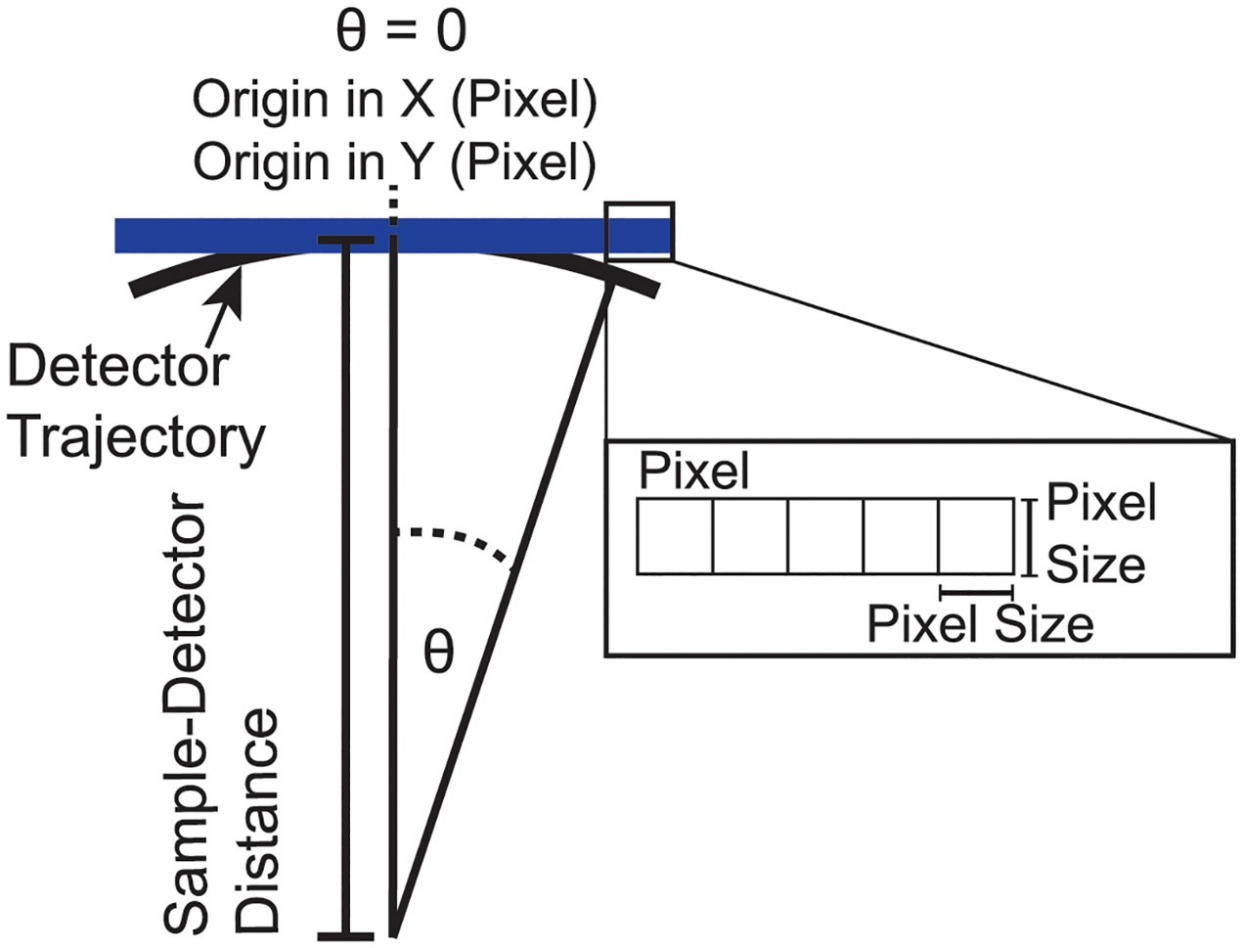
Most 2-dimensional flat detectors subtend the spherical coordinate system of the reciprocal space spanned by the angle *θ*.

Subsequently, the meridional and azimuthal angle of the diffraction experiment will be referred to as *θ* and Ξ. Most 2-dimensional flat detectors subtend the spherical coordinate system of the reciprocal space spanned by *θ* and Ξ and consequently measure a distorted image (see Fig 3). This distortion can be corrected when taking the geometry of the X-ray instrument and the data acquisition by the detector into account. However, MEDUSA allows the selective enabling and disabling this distortion correction to accommodate for specialized detectors.

Let us first discuss pixels located at *q*_‖_ = 0 Å^−1^. The azimuth angle *θ*_*k*_ for every pixel is determined by

$$\begin{array}{c} \theta_{k}=\{\begin{array}{cc} \frac{PX_{size}k}{L}\frac{360}{2\pi } & \text{non-distorted}\\ {\text{tan}}^{-1}(\frac{\left(PX_{k}-PX_{0}\right)PX_{size}}{L})\frac{360}{2\pi }, & \text{distorted} \end{array} \end{array}$$ (9)

where *PX*_*size*_ is the size of a single pixel, *PX*_*k*_ is the *k*-th pixel and *PX*_0_ is the location of the direct beam on the detector. In the same way,

$$\begin{array}{c} \Xi_{k}=\{\begin{array}{cc} \frac{PX_{size}k}{L}\frac{360}{2\pi } & \text{non-distorted}\\ {\text{tan}}^{-1}(\frac{\left(PX_{k}-PX_{0}\right)PX_{size}}{L})\frac{360}{2\pi }, & \text{distorted}. \end{array} \end{array}$$ (10)

The out-of-plane and in-plane component of the scattering vector are subsequently calculated using

$$\begin{array}{c} q_{z}^{k}=\frac{4\pi \;\text{sin}(\theta_{k})}{\lambda }\\ q_{\|}^{k}=\frac{4\pi \;\text{sin}(\Xi_{k}/2)}{\lambda }, \end{array}$$ (11)

once both angles are determined for a given dataset.

The 2-dimensional X-ray data are reduced in two different ways. 1. Data are cropped to a 12 pixel wide rectangular box centered at *q*_‖_ = 0 and averaged along *q*_‖_. This provides an out-of-plane, meridional, intensity scan that enables users to determine the repeat *d*-spacing of the stack. 2. Data are cropped into several rectangular boxes each centered at different values of *q*_*z*_ that are specified by the user. The box width is also specified by the user. One option then averages along *q*_*z*_ within the box. This determines what will be named one *q*_*z*_ in-plane line cut. A minimum of two such line cuts is required for determination of *K*_*C*_ and *B*. Another option provides horizontal *q*_*z*_ line cuts for each vertical pixel in the box. Finally, the 2-dimensional dataset as well as the out-of-plane and in-plane line cuts are visualized using the *plotly* graphing library.

### 2.5 Fitting

The intensities for each *q*_*z*_ line cut are simultaneously fitted to the membrane’s structure factor in Eq 2. The parameters in the fit are *K*_*C*_ and *B* and a separate multiplicative factor for each line cut to take into account the *q*_*z*_ dependence of the form factor *F*(*q*_*z*_). Calculating *S*(*q*_*z*_, *q*_‖_) is computationally challenging and the subprogram for this was thus written in C++. The algorithm was based on a previous program [12] but was modified to allow for GPU acceleration with the Compute Unified Device Architecture (CUDA) provided by the Nvidia Corporation [40]. GPU acceleration generally works by splitting the computation workload of a given problem between multiple processors.

The CUDA toolkit allows splitting of a processing job into *threads* that are grouped in *blocks*. The number of *threads* per *block* is a hardware specific quantity. The maximum number of *blocks* is independent of the hardware and is only limited by the CUDA toolkit [41]. As a result, a processing job can be split into as many *threads* as required. The effective speed gain is limited by the hardware. A single Geforce GTX-1080 TI graphics card, for instance, has 3584 physical CUDA cores and allows 1024 *threads* per *block*.

**Fig 4:**
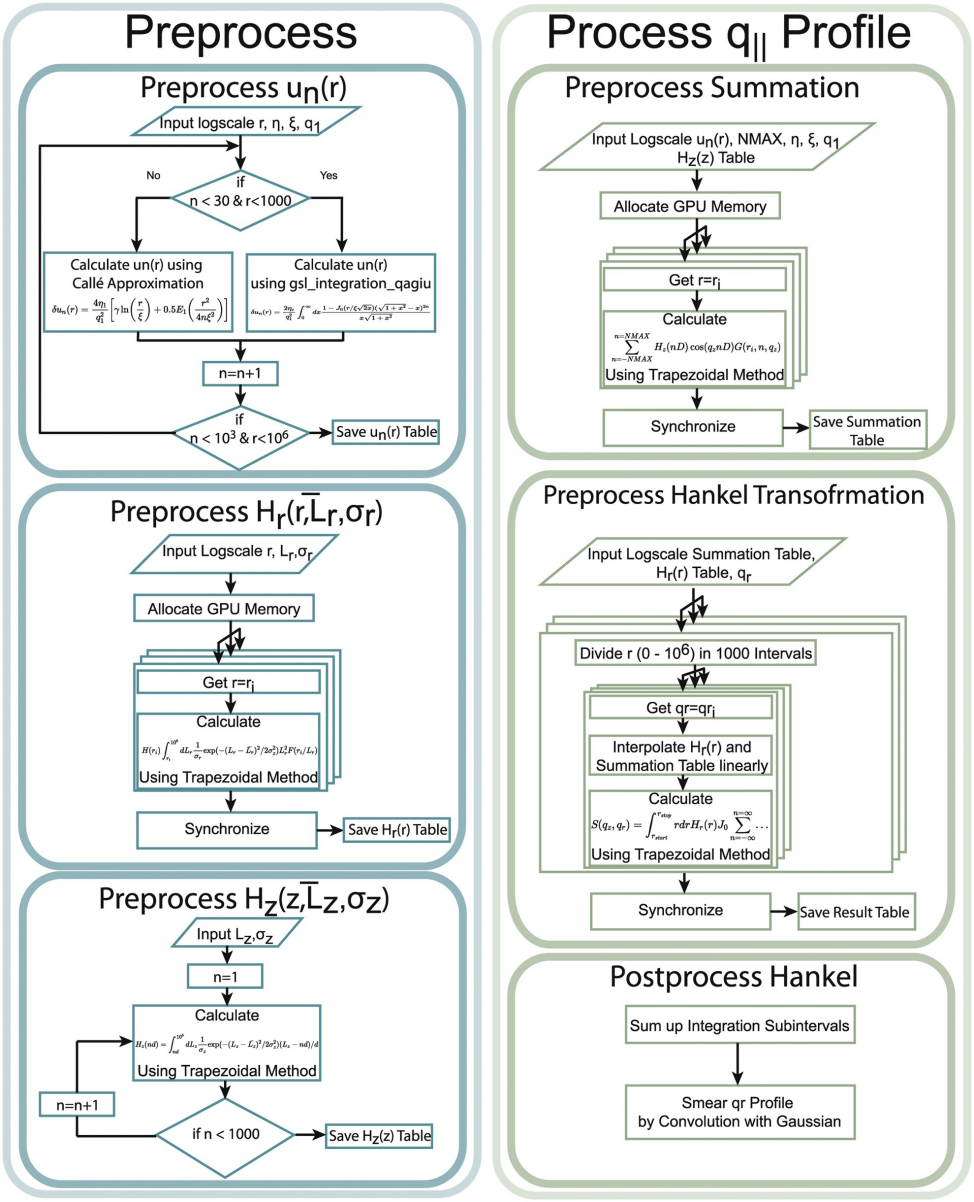
Flow diagram of the subroutines. The program pre-calculates arrays of the height-height pair correlation function *δu*_*n*_(*r*) and the finite size effect functions (Eqs 4 and 5) before calculating a single *q*_‖_ profile for given values of *q*_*z*_, *q*_‖_, *ξ*, *η* and *q*_1_.

The flow diagram of the implemented algorithm is depicted in Figs 2B and 4. The program uses the *program_options* toolbox from the *boost* *C*+ library to handle user input and can operate in two modes: It can calculate the 2-dimensional structure factor for a given *q*_*z*_ and *q*_‖_ range, or it can fit a provided data set. Both routines rely on an algorithm that calculates the structure factor for given values *q*_*z*_, *q*_‖_, *ξ*, *η* and *q*_1_. Calculating and fitting the structure factor in Eq (4) is non-trivial due to the nested integration and summation and requires computational approximations. The term that solely depends on *q*_*z*_ can be isolated from the structure factor

$$\begin{array}{c} \Lambda \left(r\right)=\sum_{n=-\infty }^{n=\infty }H_{z}\left(nD\right)\;\text{cos}\left(q_{z}nD\right)G\left(r,n,q_{z}\right)\text{,}, \end{array}$$ (12)

allowing us to rewrite Eq (4)

$$\begin{array}{ccc} S(q_{z},q_{\|}) & = & \int_{0}^{\infty }rdrH_{r}\left(r\right)J_{0}\left(q_{\|}r\right)\Lambda . \end{array}$$ (13)

We can consequently calculate Λ(*r*) only once for a given *q*_*z*_ before solving the integration in Eq (13) numerically for the desired values of *q*_‖_ (hereafter referred to as *q*_‖_-profile). The functions *H*_*r*_(*r*) and *H*_*z*_(*nD*) were introduced to account for the finite size of membrane domains. This is convenient as it reduces the required range for *n* and *r* [37].

The first step in calculating Λ(*r*) is to compute the height-height pair correlation function *δu*_*n*_(*r*). It is computationally useful to use Eq (7) for *n* < 30 and *r* < 1000 Å and employ the approximation [12]

$$\begin{array}{c} \delta u_{n}\left(r\right)=\frac{4\eta_{1}}{q_{1}^{2}}[\gamma \;\text{ln}\;(\frac{r}{\xi })+0.5E_{1}(\frac{r^{2}}{4n\xi^{2}})] \end{array}$$ (14)

for all other values for *n* and *r*. Both equations for *δu*_*n*_(*r*) are independent of the scattering vector $\vec{q}$. We can introduce the transformation $r=\bar{r}\xi$, where $\bar{r}$ is the radius for *ξ* = 1 and *η*=1. Any other combination of (*ξ*, *η*) can then be calculated by simply rescaling *r*:

$$\begin{array}{c} \delta u_{n}\left(r,\xi ,\eta \right)=\eta \delta u_{n}\left(\xi \bar{r},1,1\right). \end{array}$$ (15)

This enables us to calculate an array of *δu*(*r*, 1, 1) at the beginning of the algorithm for logarithmically spaced floating point values 10^−4^ Å< *r* < 10^6^ Å and linearly spaced integer values 0 < *n* < 1000. Eq (15) is then applied to calculate an array for *δu*(*r*, *ξ*, *η*). The integration in Eq (7) is performed numerically using the adaptive.

*gsl_integration_qagiu* algorithm provided by the GNU scientific library [42]. The trapezoidal rule can not be used due to the apparent singularity in the integrand in Eq (7) (*x* → 0).

In the same way, arrays for *H*_*r*_(*r*) and *H*_*z*_(*nd*) (see Eqs (4) and (5)) are pre-calculated. Again, logarithmically spaced floating-point values 10^−4^ Å< *r* < 10^6^ Å (10,000 values in total) and linearly spaced integer values 0 < *n* < 1000 were used in the calculation. The calculation of *H*_*r*_(*r*) is further accelerated using the CUDA toolkit by splitting the process into 10 *blocks* with 1024 *threads* each. Each *thread* then calculates the integration in Eq (5) for fixed values *r* and *n* and stores the results in an array.

In the next step, the algorithm calculates Λ(*r*) (Eq (12)) for integer values of −1000 < *n* < 1000 using the array entries from all predetermined functions. This process is split into 2 *blocks* with 1024 *threads* each. Each *thread* solely calculates the summation in Eq (12) for a given value *r* and stores the results in an array. Finally, the program calculates Eq (13). The numerical integration is performed using the trapezoidal rule with 1 Å< *r* < 10^6^ Å and a step size of 1 Å. Values of Λ between the grid points of the predetermined arrays are determined from cubic interpolation. This process is once again parallelized. Two *blocks* with 1024 *threads* each are defined, where each *thread* is instructed to compute the integration for a fixed value *q*_*r*_.

Eq (4) represents the structure factor for a finite membrane stack, but does not account for characteristics of the X-ray instrument. In a real-world experiment, the structure factor is convoluted with the beam’s footprint on the sample. The beam profile in the described setup is circular with a Gaussian distribution with a a standard deviation of *σ*_*q*_ specified by the user in both spatial directions. The determined *q*_‖_ profile is thus convoluted with a Gaussian distribution to account for this beam geometry. Multiple *q*_‖_-profiles are calculated by looping through multiple *q*_*z*_ to calculate a 2-dimensional scan of the structure factor *S*(*q*_*z*_, *q*_‖_).

The bending modulus *κ* and the membrane interaction modulus *B* can be determined independently from XDS data by fitting the *q*_‖_ dependence of the calculated structure factor *S*(*q*_*z*_, *q*_‖_) at more than one independent values of $q_{z}=q_{z}^{\left(1\right)}$ and $q_{z}=q_{z}^{\left(2\right)}$ to the experimental data. For this purpose, a Levenberg-Marquardt least squares fit was implemented using the *gsl_multimin_fminimizer* from the GNU Scientific library [42].

The function to be minimized is given by the sum of the squared residuals

$$\begin{array}{ccc} \chi^{2} & = & \sum_{l=1}^{l_{max}}\chi^{2}\left(q_{z}^{\left(k\right)}\right) \end{array}$$ (16)

$$\begin{array}{ccc} & = & \sum_{l=1}^{l_{max}}\sum_{k=1}^{k_{max}}\frac{{\left(Y_{l}\left[k\right]-y_{l}\left[k\right]\right)}^{2}}{\sigma_{l}\left[k\right]}, \end{array}$$ (17)

where *Y*_*l*_[*k*] is the interpolated value of *S*(*q*_*z*_, *q*_‖_) at discrete values $q_{z}^{\left(l\right)}$ and $q_{r}^{\left(k\right)}$ and *y*_*l*_[*k*] are the corresponding experimental values. *σ*_*l*_[*k*] are the experimental errors.

## 3 Results

### 3.1 Workflow

#### User authentication

MEDUSA requires a user authentication via the GenApp interface [38]. The login can be accessed through the link in the upper right corner and requires the user-id and the password. New users can register by clicking on the avatar icon and entering a user-id, a password (minimum 10 characters) and an email address. This authentication requirement is motivated by two considerations: First, it reduces the risk of unauthorized access and protects computational resources. Second, the data structure of GenApp differs when authentication is enabled. GenApp creates a user-specific folder to execute and store all related data within a module. This folder is set to be constant for all modules. This allows a convenient communication between the reduction and fitting module (see below). Additionally, it enables users to store fit results and revisit previous fits.

#### Reduction tab

**Fig 5:**
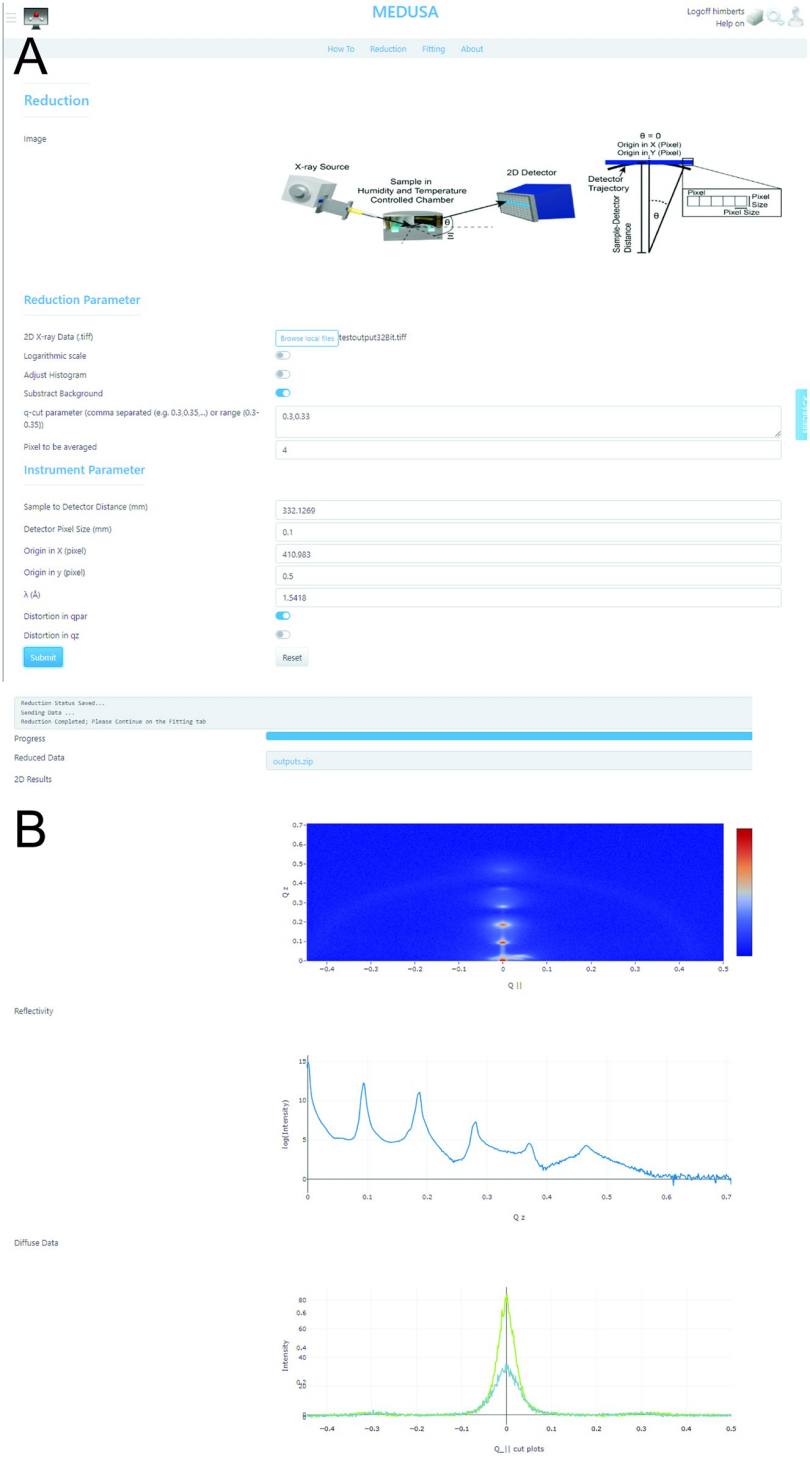
Screenshots of the reduction tab GUI: **A** Reduction-specific and Instrument-specific parameters can be entered by the user. **B** The progress of the reduction is visualized in a text-field and progress-bar. Once complete, the program visualizes the 2-dimensional intensity maps, the reflectivity profile as well as the *q*_‖_ line cuts (source: https://medusa.genapp.rocks/medusa/).

Data reduction is the first step in the analysis. It can be accessed through the reduction tab in the GenApp interface (see Fig 5). The user is asked to provide several reduction and instrumental parameter that are detailed in Table 1. A .*tiff* file can be uploaded to the server. Optionally, the user can specify whether the submitted data are on a logarithmic scale. If selected, the algorithm first converts the intensity values into a linear scale. This option consequently effects the analysis. Depending on the detector’s dynamic range, the histogram can be adjusted. This does not affect the data itself but the visualization. As discussed above, a rudimentary background subtraction routine is provided and can be optionally enabled.

**Table 1:** A summary and description of the input parameter.

**Reduction Parameters**	
2D X-ray Data (.*tiff*)	Single file containing 2-dimensional scatting data in the .*tiff* format
*q*-cut parameter	Specification out-plane scattering vector for each *q*_‖_ line-cut.
Pixel to be averaged	Specification of the box width for averaging along *q*_*z*_ in every line-cut.
**Instrument Specific Parameters**	
Sample to Detector Distance	Distance between sample and detector in mm.
	Required to calculate the accurate *q*-space of the data-set
Detector Pixel Size	Dimension of individual pixels (CCD Detector or single photon counter) in mm.
	Required to calculate the accurate q-space of the data-set
Origin in *X* and *Y*	Position of the direct beam on the detector measured in pixel.
λ	Wavelength of the instrument
Distortion in *q*_*par*_ or *q*_*z*_	Enable to apply a distortion correction in *q*_‖_ and *q*_*z*_
**Fitting Parameters**	
*ξ*	Calle parameter *ξ* in units of Å
*η*	Unitless Calle parameter *η*
Domain Size *L*_*r*_	Size of coherent scattering domains in Å.
Domain Size spread *s*_*r*_	Size-spread of coherent scattering domains in Å.
Beam Width	Width of a Gaussian beam profile in Å^−1^.

Next, the user is asked to provide *q*_*z*_ values to specify the location of the fitted line-cuts. The individual values need to be separated by a comma. Optionally a range is provided (by separating the values with the dash symbol). The algorithm then creates cuts for every available pixel in the provided range. Optionally, the intensity can be averaged over multiple pixel as specified by the *Pixel to average* entry. The data reduction is dependent on instrumental parameter as discussed above. Consequently a number of geometrical information is required but should be accessible for every instrument and careful conducted experiment. This includes the distance *L* between the detector and the sample, as well as pixel size. The position of the direct beam (*q*_*z*_=0, *q*_‖_=0) in the pixel space of the detector need to be specified as well as the X-ray wavelength λ in units of Å. The aforementioned distortion that is caused by the flat detector can be optionally corrected for either axis.

Once all parameter are provided, the user can press the highlighted submit button. This starts the process. The algorithm processes each step shown in Fig 2A. The progress can be monitored in a text-field and a progress-bar. The process typically takes 10-20 seconds to be completed. Once complete the user may download the intermediate reduction results as compressed .*zip* file. Additionally, the 2-dimensional data, the reflectivity as well as the out-of-plane profiles are visualized (see Fig 5B).

#### Fitting tab

**Fig 6:**
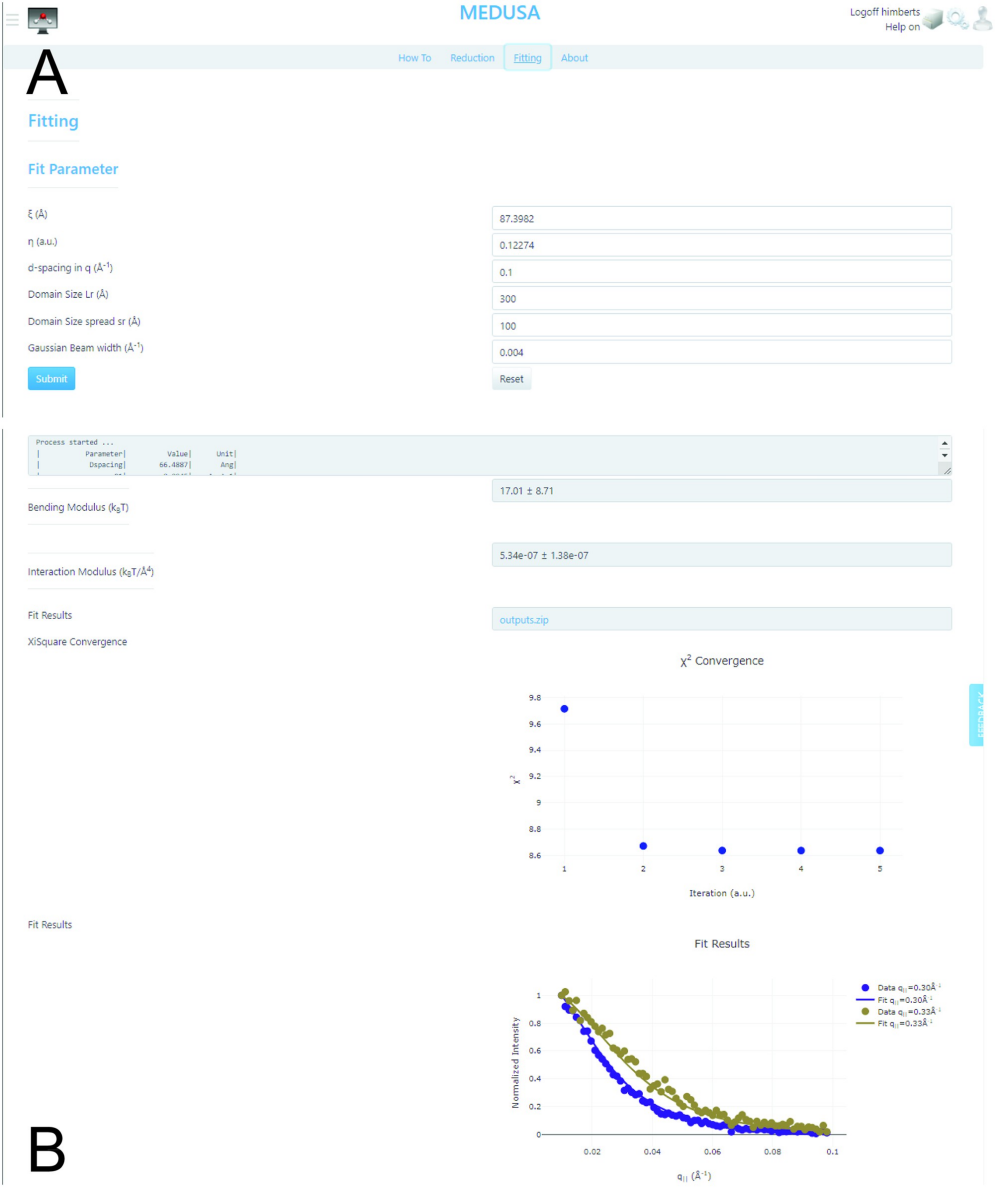
Screenshots of the fitting tab GUI: **A** Fitting-specific parameters can be entered by the user. **B** The progress of the fitting is visualized in a text-field reporting the log-output from the GPU-accelerated program. The determined bending rigidity *K*_*c*_, the interaction modulus *B* as well as *χ*^2^ are updated for every iteration. Once complete, the program visualizes the *q*_‖_ line cuts with the respective best-fit line and provides a download link to the reduced and fitted data (source: https://medusa.genapp.rocks/medusa/).

The data download in the reduction tab is optional and is not required for the fitting. The fitting tab can be selected after the reduction is completed (see Fig 6A). The fitting uses a levenberg-marquardt algorithm and requires start-parameter for the two Calle-parameter *ξ* and *η*. Fixed parameter are the membrane’s d-spacing, the domain sizes *L*_*r*_ the domain size spread *s*_*r*_ as well as the width of the Gaussian beam profile. See the discussion above for details about these parameters.

Once submitted the process starts. The log-information of the GPU-based fitting program is outputted into a text-prompt. A life-status is provided, once the algorithm reaches the least-square part of the algorithm. The bending modulus *κ* as well as the interaction modulus *B* are updated for every iteration of the algorithm together with a visualizing of *χ*^2^ (see Fig 6B). Once the fitting has converged, fits to every *q*_‖_ profile are visualized and the fit results can be downloaded as compressed .*zip* folder. This folder contains the raw-data, any graph from the GenApp interface exported as .*png* image, the reduced data as well as the individual fit results in and ascii encoded file format. Additionally, a Jupyter notebook is provided that demonstrates the visualization of the fit results for further use.

### Experimental setups

Experiments can be performed using either a synchrotron source or an in-house rotating anode machine. Data should be recorded far enough in the *q*_*z*_ direction to obtain the lamellar repeat spacing *d* and to see robust diffusion scattering. A typical range is for *q*_*z*_ from 0 to 0.5 Å^−1^. Data should also be recorded far enough on both sides of the meridian to include the full decay of the diffuse scattering in the *q*_‖_ direction in order to allow adequate background subtraction. A typical range is for *q*_‖_ from -0.5 to +0.5 Å^−1^. Two setups have been routinely used.

**Fig 7:**
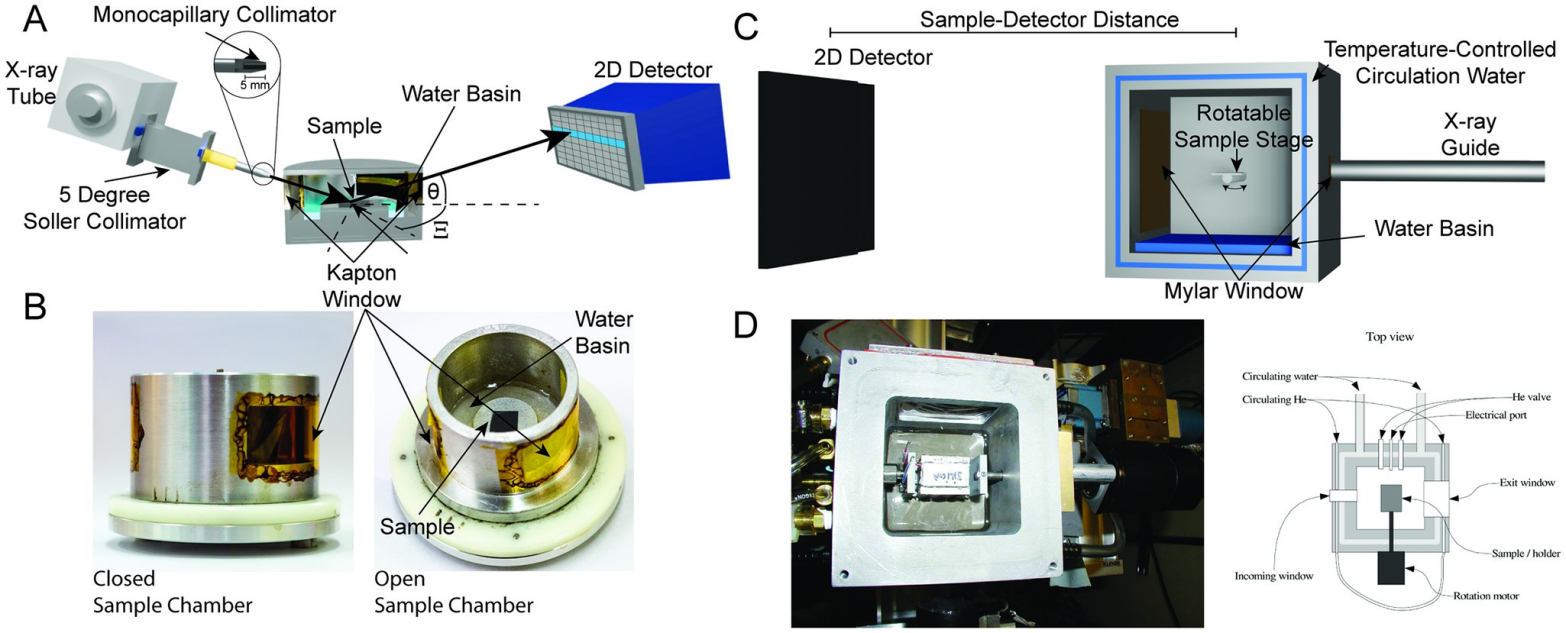
The setup of the X-ray diffraction machine is schematically sketched. The central components: X-ray tube, collimator optics, humidity chamber and detector are marked in the graphics.

1) A RIGAKU SmartLab Diffractometer. The primary components are sketched in Fig 7B. The instrument is equipped with a 9 kW CuK*α* rotating anode tube and a RIGAKU HyPix-3000 2-dimensional semiconductor detector. Multilayer optics consisting of a focusing mirror, a 5 degree soller collimator, and a 5 mm monocapillary collimator provide a circular beam with a diameter of ≈200 *μ*m, a divergence of 0.008 rad and an intensity of 10^8^ counts/mm^2⋅^s. The wavelength is λ = 1.5418 Å with a spread of $\frac{\Delta \lambda }{\lambda }=1\%$. The detector has an array of 775×385 pixels, each measuring 100×100 *μ*m^2^. Each pixel is a single photon counter with a bit-depth of 32 bit. A beam-block was installed to attenuate the intensity from the direct, *i.e*. non-scattered, beam.

2) The experiments have been performed on a synchrotron source and on standard rotating anode sources using a different setup. The incident angle is controlled by rocking the sample rather than changing the angle of the incoming synchrotron beam, and the detector has been placed at a fixed position in space. Instead of taking multiple exposures for every sample angle, every pixel of the detector accumulates counts during the entire sample rotation. Importantly, while convenient in improving the signal-to noise ratio, that type of rocking leads to an additional convolution to the data analysis to account for the pixel intensity originating from different values of *q*. Although the MEDUSA software is not written specifically for that setup, when applied to data obtained that way, MEDUSA returns values of the moduli that agree quite well with what was obtained with software [37] specifically written for that setup.

For either X-ray optical configuration a critical component in the experimental setup is a sample chamber capable of achieving between 99.9% and 100% RH in order to fully hydrate the stack of membranes from the vapor. This requires a chamber design that minimizes condensation and ensures a homogeneous temperature within the chamber. Three such chambers have been constructed [14, 40, 43].

Here, we describe the chamber specifically designed for the first set-up above ([40]) that shares features of the earlier chambers. It was machined in aluminum which is opaque for X-rays, so windows are machined on either side of the chamber and are sealed with a 13 *μ*m thick Kapton foil. This polymeric material was chosen for its high transmittance for X-rays and its defined background; it does have an unwanted diffraction signal at $|\vec{q}|\approx 0.45{Å}^{-1}$ (half-width at half-maximum ≈0.05 Å^−1^) which can be avoided, if desired, by using mylar instead of capton. The sample is placed on a stage in the center of the chamber. A basin at the bottom of the chamber below the sample is filled with a hydration solution. The humidity inside the chamber is controlled through the choice of salt and the salinity of the saline solution. For many synthetic membranes, ultra-pure water is advantageous as it allows reaching close to 100% RH. However, biological membranes such as RBC membranes are highly hygroscopic and can swell until they are washed off the silicon wafer, so it can be advantageous to lower the relative humidity by using a 40 mg/ml K_2_SO_4_ solution for hydration.

An alternative synchrotron sample design suggested in [14] was constructed at NIH in Bethesda. Fig 7C shows a schematic and Fig 7D is a photograph of the chamber. The nearly cubic aluminum chamber (5 × 5 × 6 inches) was designed for ease of use in demanding synchrotron experiments. Water circulates through channels in the one-inch thick walls, top, and bottom of the chamber from a temperature-controlled bath. The chamber has double windows with circulating helium in between, using thin mylar material to minimize scattering interference. The inner chamber contains water to hydrate the sample vapor and ensure thermal contact. Hydration is aided by a sponge on the chamber’s top, increasing evaporation surface area, and a Peltier cooler cooling the sample relative to the vapor. Sample holders for flat and cylindrical substrates rotate with programmable motors. The chamber is flushed with helium to minimize air scattering and sealed off. A thermocouple monitors temperature inside the chamber.

The discussed theory is not limited to X-rays but generally applies to scattering of other particles within a wavelength range between 1 and 10 Å. Especially neutrons are of interest. Humidity chambers for neutron beam-lines have been constructed out of a single piece of aluminum (by J. Katsaras) without the need for windows which make full hydration difficult. Chambers exist in most facilities. Neutron scattering in the form of Neutron Spin Echo (NSE) spectrometry has been widely used to measure the membrane’s bending rigidity. However, the established analysis has recently been challenged in complex lipid mixtures [44, 45]. If there is sufficient neutron intensity to observe diffuse scattering, this might provide novel insights into the membrane’s mechanical properties. Importantly, samples with multiplex lipid mixtures may be selectively deuterated enabling a domain-sensitive measurement of the bending rigidity.

## 4 Availability and future directions

We have develped MEDUSA (**ME**mbrane **D**iff**U**se **S**cattering **A**nalysis), a cloud-based analysis tool to determine the bending modulus, *κ*, from X-ray diffuse scattering experiments. MEDUSA uses GPU accelerated hardware and a parallelized algorithm to run the calculations efficiently. MEDUSA’s graphical user interface allows the user to upload 2-dimensional data collected from different sources, perform background subtraction and distortion corrections, select regions of interest, run the fitting procedure and output the fitted parameters, the membranes’ bending modulus *κ* and interaction modulus *B*. The tool is available at https://medusa.genapp.rocks.

## References

[1] Kirchhausen T. Bending membranes. *Nature Cell Biology* 2012, 14, 906–908. doi: 10.1038/ncb2570 22945258

[2] McMahon H.T.; Gallop J.L. Membrane curvature and mechanisms of dynamic cell membrane remodelling. *Nature* 2005, 438, 590–596. doi: 10.1038/nature04396 16319878

[3] McMahon H.T.; Boucrot E. Membrane curvature at a glance. *Journal of Cell Science* 2015, 128, 1065–1070. doi: 10.1242/jcs.114454 25774051 PMC4359918

[4] Helfrich W. Elastic properties of lipid bilayers: theory and possible experiments. *Zeitschrift für Naturforschung C* 1973, 28, 693–703. doi: 10.1515/znc-1973-11-1209 4273690

[5] Evans E.A. Bending elastic modulus of red blood cell membrane derived from buckling instability in micropipet aspiration tests. *Biophysical Journal* 1983, 43, 27–30. doi: 10.1016/S0006-3495(83)84319-7 6882860 PMC1329264

[6] Radmacher M. 4.-Measuring the elastic properties of living cells by the atomic force microscope. *Methods in Cell Biology* 2002, 68, 67–90. doi: 10.1016/S0091-679X(02)68005-7 12053741

[7] Brochard F.; Lennon J. Frequency spectrum of the flicker phenomenon in erythrocytes. *Journal de Physique* 1975, 36, 1035–1047. doi: 10.1051/jphys:0197500360110103500

[8] Zilker A.; Ziegler M.; Sackmann E. Spectral analysis of erythrocyte flickering in the 0.3–4-*μ*m^−1^ regime by microinterferometry combined with fast image processing. *Physical Review A* 1992, 46, 7998. doi: 10.1103/PhysRevA.46.7998 9908150

[9] Strey H.; Peterson M.; Sackmann E. Measurement of erythrocyte membrane elasticity by flicker eigenmode decomposition. *Biophysical Journal* 1995, 69, 478–488. doi: 10.1016/S0006-3495(95)79921-0 8527662 PMC1236273

[10] Park Y.; Best C.A.; Badizadegan K.; Dasari R.R.; Feld M.S.; Kuriabova T.; Henle M.L.; Levine A.J.; Popescu G. Measurement of red blood cell mechanics during morphological changes. *Proceedings of the National Academy of Sciences* 2010, 107, 6731–6736. doi: 10.1073/pnas.0909533107 20351261 PMC2872375

[11] Popescu G.; Ikeda T.; Goda K.; Best-Popescu C.A.; Laposata M.; Manley S.; Dasari R.R.; Badizadegan K.; Feld M.S. Optical measurement of cell membrane tension. *Physical Review Letters* 2006, 97, 218101. doi: 10.1103/PhysRevLett.97.218101 17155774

[12] Lyatskaya Y.; Liu Y.; Tristram-Nagle S.; Katsaras J.; Nagle J.F. Method for obtaining structure and interactions from oriented lipid bilayers. *Physical Review E* 2000, 63, 011907. doi: 10.1103/PhysRevE.63.011907 11304287 PMC2738870

[13] Liu Y.; Nagle J.F. Diffuse scattering provides material parameters and electron density profiles of biomembranes. *Physical Review E* 2004, 69, 040901. doi: 10.1103/PhysRevE.69.040901 15169001 PMC2761748

[14] Kučerka N.; Liu Y.; Chu N.; Petrache H.I.; Tristram-Nagle S.; Nagle J.F. Structure of Fully Hydrated Fluid Phase DMPC and DLPC Lipid Bilayers Using X-Ray Scattering from Oriented Multilamellar Arrays and from Unilamellar Vesicles. *Biophysical Journal* 2005, 88, 2626–2637. doi: 10.1529/biophysj.104.056606 15665131 PMC1305359

[15] Himbert S.; D’Alessandro A.; Qadri S.M.; Majcher M.J.; Hoare T.; Sheffield W.P.; Nagao M.; Nagle J.F.; Rheinstädter M.C. The Bending of the Red Blood Cell Cytoplasmic Membrane. *PLOS ONE* 2022, 17, e0269619. doi: 10.1371/journal.pone.0269619 35913930 PMC9342732

[16] Himbert S.; Qadri S.M.; Sheffield W.P.; Schubert P.; D’Alessandro A.; Rheinstädter M.C. Blood bank storage of red blood cells increases RBC cytoplasmic membrane order and bending rigidity. *PLOS ONE* 2021, 16, e0259267. doi: 10.1371/journal.pone.0259267 34767588 PMC8589153

[17] Nagao M.; Kelley E.G.; Ashkar R.; Bradbury R.; Butler P.D. Probing elastic and viscous properties of phospholipid bilayers using neutron spin echo spectroscopy. *The Journal of Physical Chemistry Letters* 2017, 8, 4679–4684. doi: 10.1021/acs.jpclett.7b01830 28892394

[18] Brown M.F. Theory of spin-lattice relaxation in lipid bilayers and biological membranes. 2H and 14N quadrupolar relaxation. *The Journal of Chemical Physics* 1982, 77, 1576–1599. doi: 10.1063/1.443940

[19] Hu M.; Diggins P. IV; Deserno M. Determining the bending modulus of a lipid membrane by simulating buckling. *The Journal of Chemical Physics* 2013, 138, 214110. doi: 10.1063/1.4808077 23758361

[20] Nagle J.F.; Tristram-Nagle S. Structure of lipid bilayers. *Biochim. Biophys. Acta* 2000, 1469, 159–195. doi: 10.1016/s0304-4157(00)00016-2 11063882 PMC2747654

[21] Shafieenezhad A.; Mitra S.; Wassall S.R.; Tristram-Nagle S.; Nagle J.F.; Petrache H.I. Location of dopamine in lipid bilayers and its relevance to neuromodulator function. *Biophysical Journal* 2023, 122, 1118–1129. doi: 10.1016/j.bpj.2023.02.016 36804668 PMC10111280

[22] Gastaldo I.P.; Himbert S.; Ram U.; Rheinstädter M.C. The Effects of Resveratrol, Caffeine, *β*-Carotene, and Epigallocatechin Gallate (EGCG) on Amyloid-*β* 25–35 Aggregation in Synthetic Brain Membranes. *Molecular Nutrition & Food Research* 2020, 64, 2000632. doi: 10.1002/mnfr.202000632 32981185

[23] Zou X.; Himbert S.; Dujardin A.; Juhasz J.; Ros S.; Stover H.D.; Rheinstadter M.C. Curcumin and homotaurine suppress amyloid-*β*25–35 aggregation in synthetic brain membranes. *ACS Chemical Neuroscience* 2021, 12, 1395–1405. doi: 10.1021/acschemneuro.1c00057 33826295

[24] Bider R.C.; Lluka T.; Himbert S.; Khondker A.; Qadri S.M.; Sheffield W.P.; Rheinstadter M.C. Stabilization of lipid membranes through partitioning of the blood bag plasticizer di-2-ethylhexyl phthalate (DEHP). *Langmuir* 2020, 36, 11899–11907. doi: 10.1021/acs.langmuir.0c01964 32903014

[25] Himbert S.; Alsop R.J.; Rose M.; Hertz L.; Dhaliwal A.; Moran-Mirabal J.M.; Verschoor C.P.; Bowdish D.M.E.; Kaestner L.; Wagner C.; et al. The Molecular Structure of Human Red Blood Cell Membranes from Highly Oriented, Solid Supported Multi-Lamellar Membranes. *Scientific Reports* 2017, 7, 39661. doi: 10.1038/srep39661 28045119 PMC5206716

[26] Himbert S.; Blacker M.J.; Kihm A.; Pauli Q.; Khondker A.; Yang K.; Sinjari S.; Johnson M.; Juhasz J.; Wagner C.; et al. Hybrid Erythrocyte Liposomes: Functionalized Red Blood Cell Membranes for Molecule Encapsulation. *Advanced Biosystems* 2020, 4, 1900185. doi: 10.1002/adbi.202070031 32293142

[27] Kučerka N.; Tristram-Nagle S.; Nagle J.F. Structure of fully hydrated fluid phase lipid bilayers with monounsaturated chains. *The Journal of Membrane Biology* 2006, 208, 193–202. doi: 10.1007/s00232-005-7006-8 16604469

[28] Tristram-Nagle S.; Nagle J.F. HIV-1 fusion peptide decreases bending energy and promotes curved fusion intermediates. *Biophysical Journal* 2007, 93, 2048–2055. doi: 10.1529/biophysj.107.109181 17526585 PMC1959562

[29] Pan J.; Tristram-Nagle S.; Nagle J.F. Effect of cholesterol on structural and mechanical properties of membranes depends on lipid chain saturation. *Physical Review E* 2009, 80, 021931. doi: 10.1103/PhysRevE.80.021931 19792175 PMC2756665

[30] Nagle J.F.; Jablin M.S.; Tristram-Nagle S.; Akabori K. What are the true values of the bending modulus of simple lipid bilayers? *Chemistry and Physics of Lipids* 2015, 185, 3–10. doi: 10.1016/j.chemphyslip.2014.04.003 24746555 PMC4199938

[31] Himbert S.; Rheinstädter M.C. Structural and mechanical properties of the red blood cell’s cytoplasmic membrane seen through the lens of biophysics. *Frontiers in Physiology* 2022, 13, 953257. doi: 10.3389/fphys.2022.953257 36171967 PMC9510598

[32] Himbert S.; Rheinstädter M.C. Erythro-VLPs: Anchoring SARS-CoV-2 spike proteins in erythrocyte liposomes *PLOS ONE* 2022, 17, e0263671. doi: 10.1371/journal.pone.0263671 35275926 PMC8916654

[33] Bose R. JC.; Kessinger C. W.; Dhammu T.; Singh T.; Shealy M. W.; Ha K.; Collandra R.; Himbert S.; Garcia F. J.; Oleinik N.; Xu B.; Vikas; Kontaridis M. I.; Rheinstädter M. C.; Ogretmen B.; Menick D. R.; McCarthy D. R. Biomimetic Nanomaterials for The Immunomodulation of The Cardiosplenic Axis Post-Myocardial Infarction *Advanced Materials* 2023, 2304615. doi: 10.1002/adma.202304615 37934471 PMC10922695

[34] Tardieu A.; Luzzati V.; Reman F. Structure and polymorphism of the hydrocarbon chains of lipids: a study of lecithin-water phases. *Journal of molecular biology* 1973, 75, 711–733. doi: 10.1016/0022-2836(73)90303-3 4738730

[35] Lei N.; Safinya C.; Bruinsma R. Discrete harmonic model for stacked membranes: theory and experiment. *Journal de Physique II* 1995, 5, 1155–1163. doi: 10.1051/jp2:1995174

[36] Rheinstädter M.C.; Häussler W.; Salditt T. Dispersion Relation of Lipid Membrane Shape fluctuations by Neutron Spin-Echo Spectrometry. *Physical Review Letters* 2006, 97, 048103. doi: 10.1103/PhysRevLett.97.048103 16907615

[37] Liu, Y. New Method To Obtain Structure Of Biomembranes Using Diffuse X-Ray Scattering: Application To Fluid Phase Dopc Lipid Bilayers. PhD thesis, Carnegie Mellon University, Pittsburg, Pennsylvania, USA, 2003. Advisor Dr. John Nagle.

[38] Savelyev A.; Brookes E. GenApp: Extensible tool for rapid generation of web and native GUI applications. Future Generation Computer Systems 2019, 94, 929–936. doi: 10.1016/j.future.2017.09.069

[39] Van Rossum G.; Drake F.L. *Python 3 Reference Manual*; CreateSpace: Scotts Valley, CA, 2009.

[40] Himbert, S. Biophysics of Blood Membranes. PhD thesis, McMaster University, 2021. Advisor Dr. Maikel Rheinstädter. http://hdl.handle.net/11375/26995

[41] NVIDIA. CUDA, release: 10.2.89, 2021.

[42] Gough B. *GNU scientific library reference manual*; Network Theory Ltd., 2009.

[43] Katsaras J.; Tristram-Nagle S.; Liu Y.; Headrick R.; Fontes E. Mason P.; Nagle J. Clarification of the ripple phase of lecithin bilayers using fully hydrated, aligned samples. *Physical Review E* 2000, 61, 5668–5677. doi: 10.1103/PhysRevE.61.5668 11031625

[44] Nagle J.F.; Evans E.A.; Bassereau P.; Baumgart T.; Tristram-Nagle S.; Dimova R. A needless but interesting controversy. *Proceedings of the National Academy of Sciences* 2021, 118, [https://www.pnas.org/content/118/20/e2025011118.full.pdf]. 10.1073/pnas.2025011118. PMC8158009 33952693

[45] Nagle J.F. Measuring the bending modulus of lipid bilayers with cholesterol. *Physical Review E* 2021, 104, 044405. doi: 10.1103/PhysRevE.104.044405 34781561

